# Development of a Hamster Model for Chikungunya Virus Infection and Pathogenesis

**DOI:** 10.1371/journal.pone.0130150

**Published:** 2015-06-12

**Authors:** Angela M. Bosco-Lauth, Sushan Han, Airn Hartwig, Richard A. Bowen

**Affiliations:** 1 Department of Biomedical Sciences, Colorado State University, Fort Collins, Colorado, United States of America; 2 Diagnostic Medicine Laboratory and Department of Microbiology, Immunology and Pathology, Colorado State University, Fort Collins, Colorado, United States of America; Singapore Immunology Network, Agency for Science, Technology and Research (A*STAR), SINGAPORE

## Abstract

Chikungunya virus is transmitted by mosquitoes and causes severe, debilitating infectious arthritis in humans. The need for an animal model to study the disease process and evaluate potential treatments is imminent as the virus continues its spread into novel geographic locations. Golden hamsters (*Mesocricetus auratus*) are often used as outbred laboratory animal models for arboviral diseases. Here we demonstrate that hamsters inoculated with chikungunya virus developed viremia and histopathologic lesions in their limbs and joints similar to those seen in human patients. The virus disseminated rapidly and was found in every major organ, including brain, within a few days of infection. Hamsters did not manifest overt clinical signs, and the virus was generally cleared within 4 days, followed by a strong neutralizing antibody response. These results indicate that hamsters are highly susceptible to chikungunya virus infection and develop myositis and tenosynovitis similar to human patients followed by a complete recovery. This animal model may be useful for testing antiviral drugs and vaccines.

## Introduction

Chikungunya fever is an emerging infectious disease caused by chikungunya virus (CHIKV), an arthropod-borne virus in the family *Togaviridae*, genus *Alphavirus*. It is a positive-sense single-stranded RNA virus transmitted primarily by *Aedes* mosquitoes, most notably *Aedes aegypti* and more recently *Aedes albopictus*. The virus was first isolated in Tanzania in 1952 and was given the name “Chikungunya,” meaning “that which bends up” in the Makonde language [1, 2]. The name is descriptive of the often severely debilitating arthritis that can last for months following infection, although the virus is only fatal in rare cases [3, 4]. CHIKV endemic regions span from Africa throughout Southeast Asia and islands in the Indian Ocean. Within these regions, CHIKV traditionally causes cyclical epidemics approximately every 10 years with only sporadic small outbreaks in the interim [5]. Within the past decade, however, the number of CHIKV epidemics has increased dramatically, and viruses isolated from both endemic and newly affected regions are representative of old and new strains from the three distinct clades: West African, East-Central-South African, and Asian [4, 6]. Between 2001 and 2007, CHIKV outbreaks swept across the Indian Ocean, causing widespread disease with the most notable epidemic taking place on La Reunion Island where more than one third of the population was affected and more than 200 died [7]. In 2007, the first European outbreak of chikunguna took place in Italy, making it the most temperate region with an established CHIKV presence [8]. Presently, an outbreak in the Caribbean is being described as the first establishment of CHIKV in the Western Hemisphere in non-travelers. A single point mutation in the envelope glycoprotein has been associated with an increase in vector competence for *Ae*. *albopictus*, which may account for some of the increased incidence of CHIKV infections in previously naïve urban areas [9, 10]. It is suspected that CHIKV is maintained by both sylvatic and epidemic transmission cycles, and while non-human primates are susceptible and can be experimentally infected, the nature of the sylvatic cycle is largely unclear [5]. Under laboratory settings, certain strains of mice, mostly juveniles, can be experimentally infected, but it has often been difficult to demonstrate repeatability using mouse models [11, 12, 13, 14, 15]. Here we describe the use of hamsters as a small animal model for CHIKV infection and demonstrate that in response to subcutaneous inoculation of virus, they developed a high titer viremia and histopathologic lesions in joints and muscles similar to that observed in other susceptible species.

## Materials and Methods

### Ethics Statement

All experiments were approved by the Institutional Animal Care and Use Committee of Colorado State University, Fort Collins, Colorado, USA, under approval number 09-001A.

### Animals

Four to six-week-old male hamsters were obtained from Charles River Laboratories and housed in the animal biosafety level (ABSL) 3 facility at Colorado State University. Hamsters were housed 5 per cage and fed a commercial rodent feed diet.

### Viruses

Two strains of CHIKV were provided by Ann Powers at the Centers for Disease Control and Prevention, Fort Collins, CO. The SAH2123 (SAH) virus was derived from a human isolate in 1976 and had been passaged three times in Vero cells. The COM2005 (COM) virus was isolated from mosquitoes collected during the Comoros Island outbreak in 2005 and had been passaged twice in suckling mice and seven times in cultured cells. Both viruses are classified as members of the East-Central-South African clade of CHIKV.

### Experimental infection and sample collection

A pilot experiment was conducted to determine susceptibility of hamsters to CHIKV using two strains of virus. Five hamsters were infected by subcutaneous inoculation in the ventral abdomen with SAH and 5 with COM viruses, both diluted in sterile PBS to achieve an inoculation titer of 10^5^ plaque forming units (pfu) per 100μl. The hamsters were approximately six months of age at the time of inoculation. Serum samples were collected daily for the first five days after inoculation for virus titration and clinical observations were recorded through day 14, at which time hamsters were euthanized and terminal blood samples collected for serology.

For the larger pathogenesis study, 25 hamsters were acclimated for 5 days prior to infection, during which time they were weighed and joint measurements were taken of the left hock joint using microcalipers, measuring in 1/100^th^ inch increments. The SAH strain virus was diluted in sterile PBS to achieve an inoculation titer of 10^5^ pfu per 100μl. Twenty hamsters were infected with CHIKV and 5 were injected with PBS at five weeks of age. Inoculations were given subcutaneously in the left pelvic limb in the lateral thigh region. Following inoculation, hamsters were weighed daily, joint measurements of the left hock were recorded daily and blood samples collected daily for the first 6 days, then again 10, 14, and 28 days post-infection (DPI.) Blood was collected under brief isoflurane anesthesia from the cranial vena cava and serum collected following centrifugation. On days 1, 2, 3, 4, 6, 10, and 14, 2 hamsters were euthanized and necropsied, serum and organs were saved for virus isolation, and organs (heart, lungs, liver, spleen, kidney, small intestine, brain, skin, and muscle) were fixed and prepared for histopathology. Serum was diluted into BA-l medium (MEM salts, 1% bovine serum albumin, 350 mg/L sodium bicarbonate, 100 units/mL penicillin, 100 μg/mL streptomycin, 2.5 μg/mL amphotericin B in 0.05 M Tris, pH 7.6) for a working concentration of 1:10. Sera from terminal blood samples were frozen undiluted and saved for serological assay. For virus isolation, 0.1 gram fragments of each harvested organ were frozen to -80°C until assays were performed. In addition to the organs listed above, the left pelvic limb in which the inoculation was given and the right thoracic limb were removed and fixed in buffered formalin.

### Virus Titration and Serology

Virus concentrations in serum and organ homogenates were determined by plaque assay, as previously described [16]. Briefly, serial 10-fold dilutions of serum in BA-1 medium were prepared and 100 μl volumes were inoculated onto confluent monolayers of Vero cells in 6-well tissue culture plates. Samples were incubated for one hour, then overlayed with 0.5% agarose in minimal essential media (MEM) containing 2% FBS and antibiotics. Plates were incubated at 37°C for 24 hours, at which time a second overlay containing neutral red was added. Plaques were counted 48 hours after initial inoculation. For virus isolation from organs, frozen tissues were homogenized in BA-1 using a mixer mill to obtain 10% suspensions. Samples were centrifuged and the supernatants serially diluted and assayed by plaque assay as described for serum.

Plaque reduction neutralization tests (PRNT) were used to detect neutralizing antibodies in serum, as previously described [16]. Serum samples were heat-inactivated at 56°C for 30 minutes, diluted 1:5 in BA-1 medium, and serial 2-fold dilutions prepared. An equal volume of virus was added to each sample and the virus-serum mixtures incubated at 37°C for 1 hour to allow antibody to react to viral antigen. Following incubation, samples were inoculated onto 6 well plates of Vero cell monolayers and the same protocol for incubation and overlay was followed as for virus titration.

### Histopathology and Immunohistochemistry

Tissues were fixed in buffered formalin for 2 days, then transferred to 70% ethanol, prepared for histology by dehydrating in alternating xylene and alcohol baths, and embedded in paraffin wax. Sections (5 μm) were cut on a microtome and stained with hematoxylin and eosin using standard methods.

Tissue sections from uninfected and CHIKV-infected hamsters were collected onto charged slides, deparaffinized and subjected to antigen retrieval by heating to 95°C in Target Retrieval (DAKO) for 25 min followed by a 20 min cooling-down period at room temperature (rt). The sections were then subjected to the following blocking steps at rt incubation: (i) peroxidase-block (DAKO) for 10 min, (ii) 0.15M glycine in PBS for 15 min, (iii) DAKO antibody-diluent-solution for 30 min, with a brief rinse in TRIS-buffered saline with 0.1% Tween-20 (TBST) between each. The sections were then incubated for 2 hours at room temperature with a monoclonal antibody (5.5G9) specific to the capsid protein of CHIKV (Goh, et al., J Gen Virol, in press), followed by 10 min of repeated TBST-washes. Antibody binding was visualized using the anti-mouse-IgG Envision kit (DAKO) according to the manufacturer’s instructions. Sections were counterstained with Meyer’s hematoxylin, mounted with Glycergel (DAKO) and examined under a microscope.

### Statistical Analyses

Differences in weights and hock joint width over time was analyzed by repeated measures analysis of variance using SAS Proc GLM.

## Results

### Pilot study

**Fig 1:**
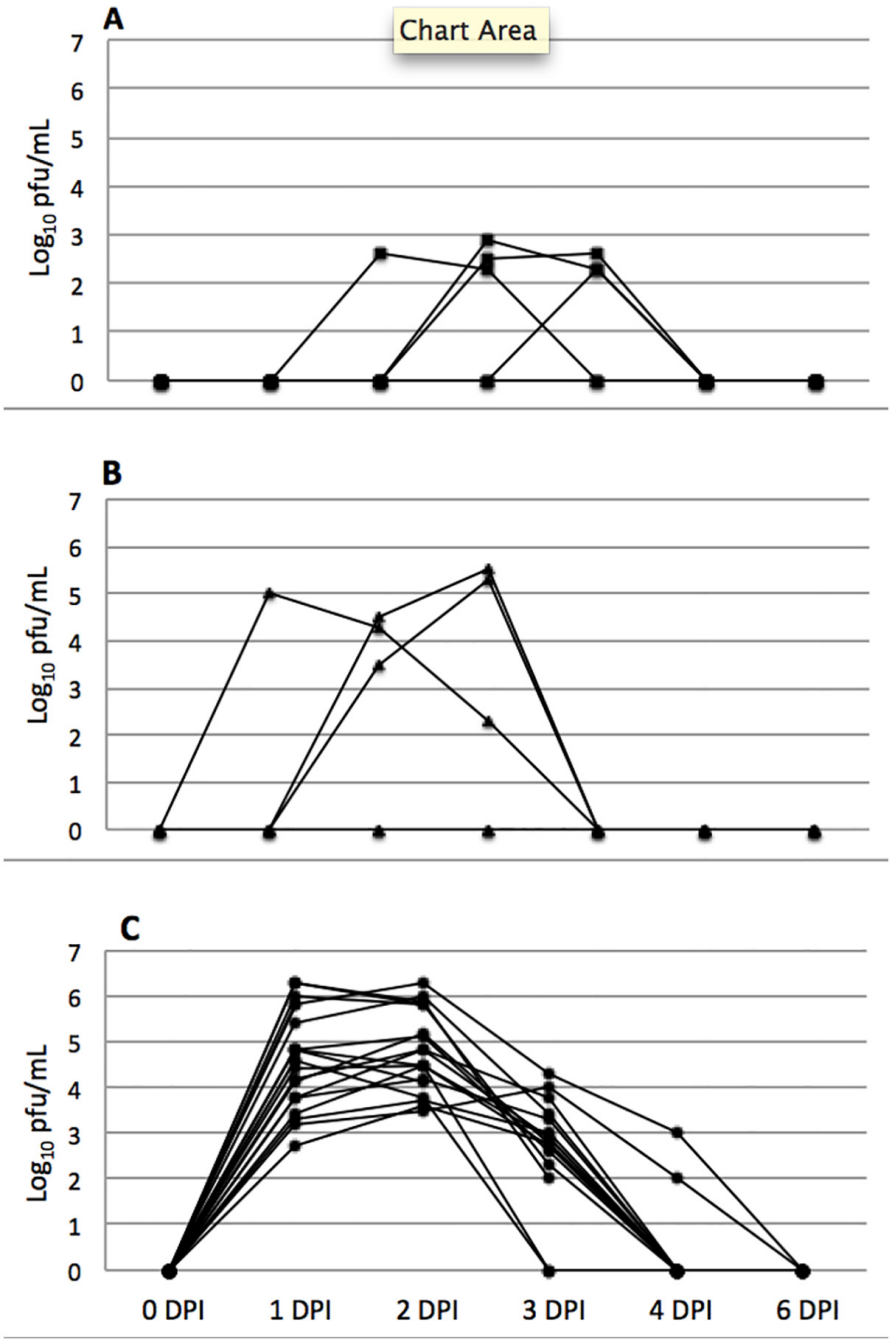
Viremia in hamsters infected with CHIKV. A) hamsters infected with COM strain virus (pilot study, n = 5); B) hamsters infected with SAH strain virus (pilot study, n = 5); C) hamsters infected with SAH strain virus (main study, n = 18). Minimum detectable viremia was 2.0 log_10_ pfu/mL; a titer of 0 indicates no virus was detected.

A total of 10 hamsters were used in the pilot study, 5 for each strain of CHIKV. Four of 5 hamsters inoculated with the COM strain developed viremia between 2 and 4 DPI, with peak titers between 2.6 and 2.9 log_10_ pfu/mL (Fig 1). For the SAH virus, 3 of 5 hamsters developed viremia between days 1–4 PI, with peak titers ranging from 5–5.6 log_10_ pfu/mL (Fig 1). None of the 10 hamsters in the pilot experiment seroconverted by 14 DPI. Based on these preliminary results, the SAH virus was used for the larger pathogenesis study, as the viremia titers peaked earlier and were higher overall. At no point did any of the hamsters exhibit behavior indicative of pain, distress, or illness.

### Clinical responses

None of the inoculated hamsters demonstrated overt clinical signs of disease, similar to what was observed in the pilot study. Several of the animals experienced slight decreases in weight during the first 6 DPI, but because the same phenomena occurred in the control animals, these weight fluctuations were attributed to handling stress and blood sample collection. There was not a significant difference in weight gain over the first 10 days of infection between control and infected hamsters (p > 0.1) The hamsters that were kept beyond day 10 post-infection all gained significant amounts of weight as would be expected for juvenile animals. Significant swelling of the hock joint was not detected using calipers in any of the control or inoculated hamsters (p > 0.1) and legs did not appear inflamed or painful at any point following virus inoculation.

### Virus Titration and Serology

Each of the hamsters inoculated with CHIKV developed viremia by 1 day post-infection (DPI), with peak titers occurring on 1 or 2 DPI and clearance of virus between 3 and 4 DPI. Peak titers ranged from 3.6–6.3 log pfu/ml (Fig 1). Viral load in organs varied depending upon time of euthanasia, but all tissues tested positive for virus isolation in those animals euthanized on 1 and 2 DPI. Virus had been cleared from the liver of both animals that were euthanized on 3 DPI, and by 4 DPI virus was cleared from the liver and heart in both animals. None of the hamsters euthanized after 4 DPI had detectable infectious virus in any organ (Table 1).

**Table 1:** Organ Virus Burdens Over Time Following Inoculation with CHIKV (SAH strain).

		Virus titer (log_10_ PFU/gram of tissue)								
Hamster	Day Euthanized	Heart	Lung	Liver	Kidney	Spleen	Brain	Skin	Muscle	Sm. Int.
1	1	5.2	6.8	7.1	5.6	5.1	4.8	4.4	4.8	4.8
2	1	5.0	4.2	5.8	4.0	4.0	<2	4.2	4	3.1
3	2	3.5	7.0	5.0	3.8	3.5	3.6	4.1	4.8	2.8
4	2	4.6	7.0	5.5	5.6	5.6	4.6	4.1	6.3	3.1
5	3	3.6	5.6	<2	2.5	2.5	4.8	4.2	4.1	2.3
6	3	<2	5.2	<2	2.0	3.0	4.0	4.4	3.3	<2
7	4	<2	4.9	<2	2.5	2.5	5.2	2.3	2.5	3.0
8	4	<2	4.6	<2	<2	2.8	3.3	<2	<2	<2
9	6	<2	<2	<2	<2	<2	<2	<2	<2	<2
10	6	<2	<2	<2	<2	<2	<2	<2	<2	<2
11	10	<2	<2	<2	<2	<2	<2	<2	<2	<2
12	10	<2	<2	<2	<2	<2	<2	<2	<2	<2
13	14	<2	<2	<2	<2	<2	<2	<2	<2	<2
14	14	<2	<2	<2	<2	<2	<2	<2	<2	<2

PRNT assays were performed only on the 6 animals euthanized at 28 DPI) at which time all had seroconverted, with neutralizing antibody titers between 40–160 using an endpoint of 80% neutralization. None of the 5 control hamsters developed detectable neutralizing antibodies.

### Histopathology and Immunohistochemistry

**Fig 2:**
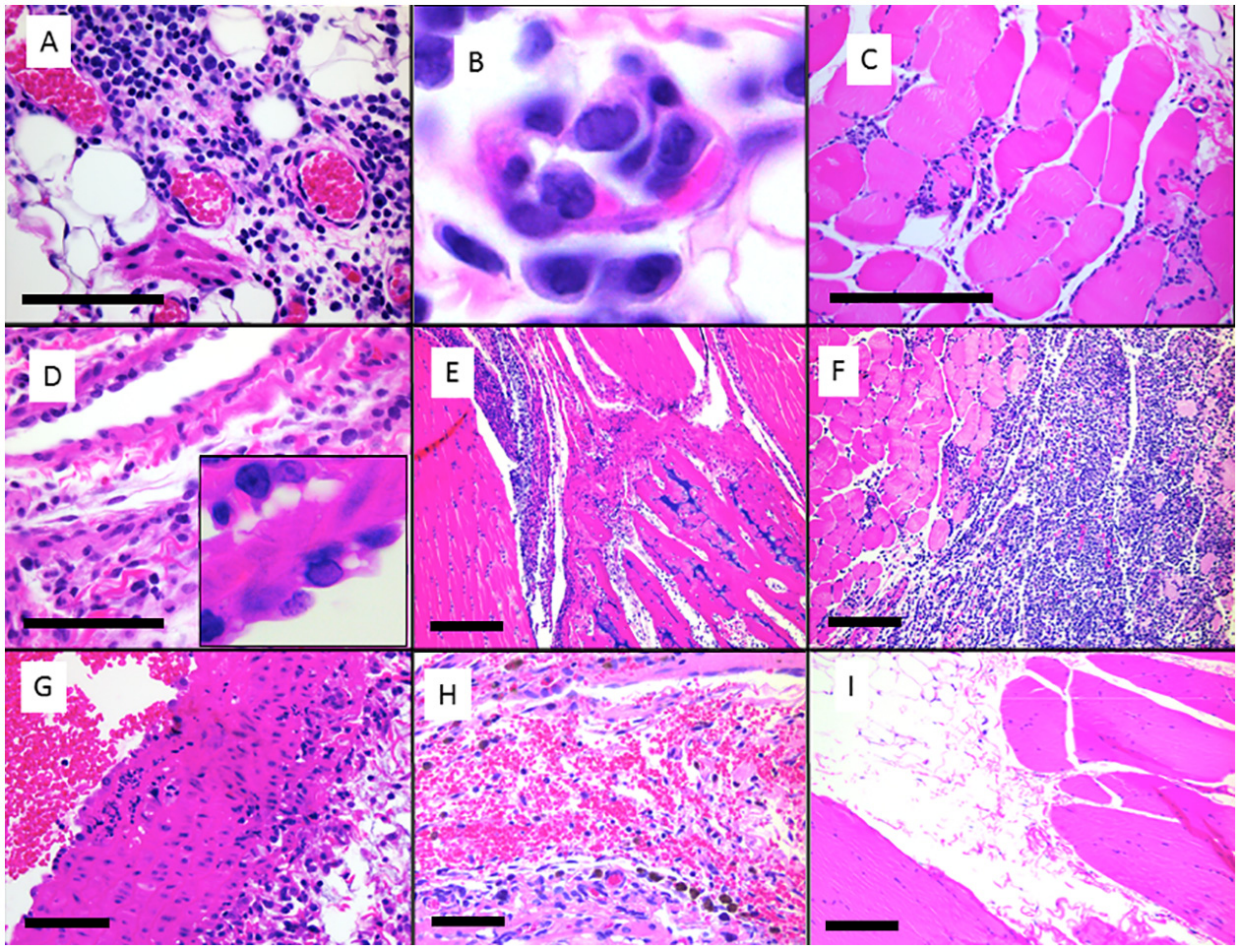
Photomicrographs of tissues from CHIKV infected golden Syrian hamsters and control hamsters. CHIKV infected hamster 5 at 3 DPI had moderate mononuclear fasciitis of the left hind limb (A) associated with vascular endothelium with occasional amphophilic intranuclear inclusion bodies (B). CHIKV infected hamster 6 at 3 DPI had necrotizing and mononuclear myositis and fasciitis of the left hind limb (C) and mononuclear epicarditis (D) with vascular endothelial inclusion bodies (inset). CHIKV infected hamster 9 at 6 DPI had mononuclear tenosynovitis of the left hindlimb (E). CHIKV infected hamster 10 at 6 DPI had severe necrotizing and mononuclear myositis of the left hindlimb (F). Chik V infected hamster 13 at 14 DPI had necrotizing vasculitis of the great vessels and associated myocarditis (G). Distal hind limbs of control hamsters inoculated with PBS and euthanized on 28 DPI had small amounts of histiocytic inflammation and hemosiderin around a blood vessel (H), with no myositis or fasciitis in skeletal muscle (I). Scale bars represent 100 μm (A, D, G) or 400 μm (C, E, F, H, I).

Tissues from 2 hamsters euthanized 3, 6 and 14 days after virus inoculation, along with tissues from 2 control hamsters euthanized at 28 days were examined histologically. At 3 DPI, both hamsters had moderate mononuclear and mastocytic inflammation and edema of the hind limb fascia and abdominal mesentery. Associated blood vessels had rare endothelial cells with possible amphophilic intranuclear inclusion bodies and margination of the chromatin (Fig 2). One of 2 hamsters additionally had mild epicarditis, myocarditis and moderate mediastinal inflammation, most likely from repeated venipuncture in that area. At 6 DPI, hind limb fasciitis varied from mild to severe. Both hamsters had necrotizing myositis of the hind or forelimb and 1 of 2 had myocarditis. At 14 DPI, one of two hamsters had severe hindlimb myositis, and both had mild tenosynovitis involving all limbs. The two control hamsters, euthanized at 28 DPI, had normal tissues with no inflammation.

**Fig 3:**
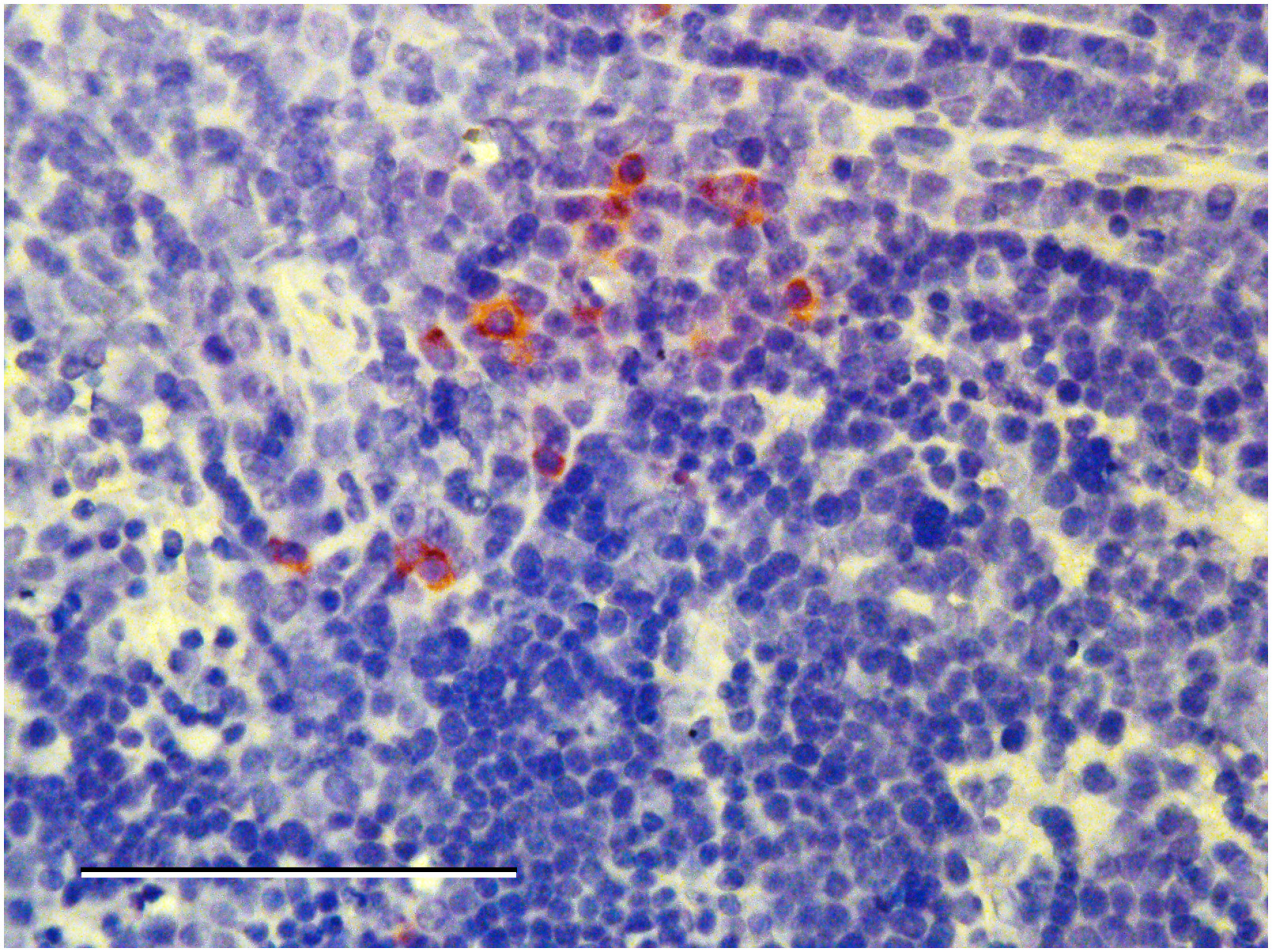
Immunohistochemistry stain of spleen from an infected hamster. ChikV antigen-positive cytoplasmic staining of mononuclear cells on day 2 post-inoculation in the spleen of a hamster infected with SAH virus. Scale bar represents 100 μm.1

Hamster tissues with inflammatory histopathologic lesions were immunohistochemically processed to detect CHIKV antigens. Staining was not detected in any of the affected tissues. However, in 1 hamster euthanized on 2 DPI, several mononuclear cells in the spleen and popliteal lymph node from the injected limb showed strong antigen-positive staining of the cytoplasm (Fig 3). Staining was not observed in tissues from the negative control animals

## Discussion

Chikungunya virus has infected and caused disease in millions of people over the past decade and its geographic range continues to expand, most recently to multiple islands in the Caribbean and into the continental U.S. Additionally, chikungunya fever and dengue fever often coincide and the actual incidence of CHIKV infection in most areas is unknown but likely under-recognized [17]. There is currently no effective vaccine or direct treatment for the disease, leaving vector control and repellents as the only effective strategies to combat this disease. In order to develop vaccines or therapeutics, scientists need the ability to first understand viral pathogenesis in a host and then test potential treatments, all of which require an effective animal model.

An animal model useful for testing countermeasures to human arbovirus infections should develop a viremia capable of infecting feeding mosquitoes and develop disease with clinical and/or pathologic similarities to that observed in people. Primates provide such a model in that they develop high viremia titers and signs similar to those reported for human disease cases [18, 19], but their use is limited by high cost and relative lack of approved institutions that can support primate research. Ideally, small rodent models could provide researchers with ample animal numbers and cost efficiency, and rodents can be easily housed in most ABSL2/3 laboratories. Previous attempts at using mice for models of CHIKV infection have identified certain laboratory strains, including interferon knockout mice and neonatal C57BL/6 mice as potential disease models, although the virus replicates poorly or not at all in many laboratory mouse strains [11, 12]. Hamsters make excellent lab animal models for other arboviruses, such as yellow fever virus, West Nile virus and Japanese encephalitis virus, and are outbred rodents with an intact immune system [20, 21, 22].

*Ae*. *aegypti and Ae*. *albopictus* are the primary mosquito vectors of CHIKV [23], and both are catholic feeders with urban habitats and preference for human and other mammalian bloodmeals [24]. For transmission from an infected host to a competent vector, the host must have sufficiently high viremia for the mosquito to ingest an infectious dose of virus and develop a disseminated infection. For *Ae*. *albopictus* and *Ae*. *aegypti* respectively, greater than 50% of mosquitoes were infected after feeding on blood meals containing 10^3.6^ and 10^5.0^ pfu/mL of blood [25]. In this study, we demonstrated that a majority of inoculated hamsters developed a viremia that exceeded the experimental mosquito infectivity threshold, indicating that they may be useful in vector-vertebrate models of CHIKV infection. Experimental studies with mice revealed that in juvenile animals, the peak viremia was equivalent or less than the titers we found in hamsters [12].

The second important finding of this study relative to animal model development is that hamsters develop significant inflammatory lesions involving skeletal muscle, fascia and tendon sheaths of multiple limbs following infection with CHIKV. This mimics the disease in humans and may lead to useful information about viral mechanisms of pathogenesis in an immune-competent host. Previous studies using mice have also been able to replicate arthralgia, either with interferon-deficient or neonatal mice [12, 26]. One study using adult C57BL/6 mice demonstrated similar findings [27]. Our study indicates that outbred hamsters can be infected with wild-type CHIKV from a historical East-Central-South African clade and develop consistent infections without passaging the virus through the host. Unlike reports using mouse models, we failed to detect overt clinical disease in infected hamsters, but widespread inflammatory response was readily demonstrated by histopathology. Furthermore, the presence of CHIKV infected cells in lymphatic tissues is consistent with what has been observed in mice [12]. In terms of vaccination, hamsters can be used as test subjects because they develop consistent viremia, which is the basis for determination of infection. Clinically, the development of inflammatory lesions in joint associated tissues may prove a very useful tool for studying therapeutics as a means of developing treatments for symptomatic patients with chikungunya fever.
